# High mitochondria content is associated with prostate cancer disease progression

**DOI:** 10.1186/1476-4598-12-145

**Published:** 2013-11-21

**Authors:** Katharina Grupp, Karolina Jedrzejewska, Maria Christina Tsourlakis, Christina Koop, Waldemar Wilczak, Meike Adam, Alexander Quaas, Guido Sauter, Ronald Simon, Jakob Robert Izbicki, Markus Graefen, Hartwig Huland, Thorsten Schlomm, Sarah Minner, Stefan Steurer

**Affiliations:** 1 General, Visceral and Thoracic Surgery Department and Clinic, University Medical Center Hamburg-Eppendorf, Hamburg, Germany; 2 Institute of Pathology, University Medical Center Hamburg-Eppendorf, Martinistr. 52, 20246 Hamburg, Germany; 3 Martini-Clinic, Prostate Cancer Center, University Medical Center Hamburg-Eppendorf, Hamburg, Germany; 4 Department of Urology, Section for translational Prostate Cancer Research, University Medical Center Hamburg-Eppendorf, Hamburg, Germany

**Keywords:** MTC02, *ERG*, Prostate cancer, Tissue microarray

## Abstract

**Background:**

Mitochondria are suggested to be important organelles for cancer initiation and promotion. This study was designed to evaluate the prognostic value of MTC02, a marker for mitochondrial content, in prostate cancer.

**Methods:**

Immunohistochemistry of using an antibody against MTC02 was performed on a tissue microarray (TMA) containing 11,152 prostate cancer specimens. Results were compared to histological phenotype, biochemical recurrence, *ERG* status and other genomic deletions by using our TMA attached molecular information.

**Results:**

Tumor cells showed stronger MTC02 expression than normal prostate epithelium. MTC02 immunostaining was found in 96.5% of 8,412 analyzable prostate cancers, including 15.4% tumors with weak, 34.6% with moderate, and 46.5% with strong expression. MTC02 expression was associated with advanced pathological tumor stage, high Gleason score, nodal metastases (*p* < 0.0001 each), positive surgical margins (*p* = 0.0005), and early PSA recurrence (*p* < 0.0001) if all cancers were jointly analyzed. Tumors harboring *ERG* fusion showed higher expression levels than those without (*p* < 0.0001). In ERG negative prostate cancers, strong MTC02 immunostaining was linked to deletions of *PTEN*, 6q15, 5q21, and early biochemical recurrence (*p* < 0.0001 each). Moreover, multiple scenarios of multivariate analyses suggested an independent association of MTC02 with prognosis in preoperative settings.

**Conclusions:**

Our study demonstrates high-level MTC02 expression in ERG negative prostate cancers harboring deletions of *PTEN*, 6q15, and 5q21. Additionally, increased MTC02 expression is a strong predictor of poor clinical outcome in ERG negative cancers, highlighting a potentially important role of elevated mitochondrial content for prostate cancer cell biology.

## Background

Prostate cancer is a major cause of cancer-related mortality and morbidity in males [1]. Although the majority of prostate cancers present as low malignant, indolent tumors, there is also an aggressive subset. For example in Germany, about 60,000 new cases of prostate cancer are diagnosed every year, and still about 11,000 patients die from their disease [2]. The common pre-operative parameters including Gleason grade, tumor extent in biopsies, and preoperative prostate specific antigen (PSA) levels are statistically powerful prognosticators, however insufficient to allow for optimal treatment decisions in individual patients. Accordingly, there is a considerable need for improved diagnostic tools to early distinguish these patients requiring aggressive therapy with all its associated side effects from the majority of patients who will not. It is hoped, that advances in basic prostate cancer research will eventually lead to novel prognostic biomarkers and better therapeutic options.

The growing interest in mitochondrial function and dysfunction reflects the potential role of mitochondria for cancer development [3]. Loss of proliferation control in cancer cells eventually results in cellular bulks that extend beyond the capacity of their vasculature, resulting in oxygen and nutrient deprivation. Accordingly, tumor growth is accompanied by cellular adaptations to overcome these limitations. Mitochondria are key organelles for energy production including glucose metabolism and oxidative phosphorylation with a critical role in cell survival and apoptosis. Amount and activity of mitochondria may hence play a critical role in tumor initiation and progression [4], and it is not surprising that mutations of mitochondrial genes or alterations of the mitochondrial content have been suggested to play an important role in various cancer types [5-8]. As a consequence, an increasing number of anti-cancer drugs is under development [9-11] targeting mitochondria and associated structures. Some studies have even suggested that intracellular accumulation of mitochondria (the so-called mitochondrion-rich phenotype) might represent an important adaptive mechanism in rectal and breast cancer [12,13].

Mutations of mitochondrial DNA were also identified in prostate cancer [14-22] and deregulated mitochondrial metabolism has been suggested to play a relevant role in prostate carcinogenesis [23-25]. Based on these reports, we hypothesized that also the quantity of mitochondria present in prostate cancer cells might be of clinical relevance, and that the cellular mitochondria content might vary between prostate cancer subgroups harboring different key molecular alterations that might influence cell metabolism.

The antibody MTC02 (mouse monoclonal to mitochondria) recognizes a 60 kDa non glycosylated protein component of mitochondria found in human cells, and has been used to determine the mitochondrial content of tumor cells in a variety of previous studies. For example, earlier studies used MTC02 to determine the molecular genetic alterations [13] and the frequency, morphology and clinical features of mitochondrion-rich breast cancers [26]. A tissue microarray (TMA) containing 11,152 prostate cancer specimens with clinical follow-up information and an attached molecular database was analyzed in order to evaluate the clinical significance of mitochondria content, and to search for possible associations with molecularly defined cancer subgroups. Our study demonstrates that “mitochondrion-rich phenotype” is strongly associated with molecular cancer features and strongly linked to poor prognosis in ERG negative prostate cancers.

## Materials and methods

### Patients

Radical prostatectomy specimens were available from 11,152 patients, undergoing surgery between 1992 and 2011 at the Department of Urology, and the Martini Clinics at the University Medical Center Hamburg-Eppendorf. Research using pseudomized human left-over tissue samples from routine diagnosis was performed in compliance with the Helsinki Declaration, and is covered by §12 of the Hamburgisches Krankenhausgesetz (HmbKHG). Manufacturing and usage of tissue microarrays for research purposes has been has been approved by the Institutional Review Board of the Aerztekammer Hamburg (Chair: Prof. T. Weber, Ref. WF-049/09). Follow-up data were available of 9,695 patients with a median follow-up of 36.8 months (range: 1 to 228 months; Table 1). Prostate specific antigen values were measured following surgery and recurrence was defined as a postoperative PSA of 0.2 ng/ml and increasing at first of appearance. All prostate specimens were analyzed according to a standard procedure, including complete embedding of the entire prostate for histological analysis [27]. The TMA manufacturing process was described earlier in detail [28]. In short, one 0.6 mm core was taken from a representative tissue block from each patient. The tissues were distributed among 24 TMA blocks, each containing 144 to 522 tumor samples. Presence or absence of cancer tissue was validated by immunohistochemical AMACR and 34BE12 analysis on adjacent TMA sections. For internal controls, each TMA block also contained various control tissues, including normal prostate tissue. The molecular database attached to this TMA contained results on ERG expression in 9,628, *ERG* break apart fluorescence *in-situ* hybridization (FISH) analysis in 6,106 (expanded from [29]), and deletion status of 5q21 in 3,037 [30], 6q15 in 3,528 (extended from [31]), *PTEN* in 6,130 [32] and 3p13 in 1,290 tumors (unpublished data) tumors.

**Table 1:** Composition of the prognostic tissue microarray containing 11,152 prostate cancer specimens

	**No. of patients**	
	**Study cohort on tissue microarray (n = 11,152)**	**Biochemical relapse among categories (n = 1,824)**
**Follow-up (mo)**		
Mean	53.4	
Median	36.8	
Age (y)		
<50	318	49
50-60	2.77	460
60-70	6.55	1.08
>70	1.44	232
**Pretreatment PSA (ng/ml)**		
<4	1.41	142
4-10	6,735	827
10-20	2,159	521
>20	720	309
**pT category (AJCC 2002)**		
pT2	7.370	570
pT3a	2.41	587
pT3b	1.26	618
pT4	63	49
**Gleason grade**		
≤3 + 3	2.86	193
3 + 4	1.57	573
4 + 3	6.18	849
≥4 + 4	482	208
**pN category**		
pN0	6.12	1.13
pN+	561	291
**Surgical margin**		
Negative	8.98	1.15
Positive	1.970	642

NOTE: Numbers do not always add up to 11,152 in the different categories because of cases with missing data. *Abbreviation*: *AJCC* American Joint Committee on Cancer.

### Immunohistochemistry

Freshly cut TMA sections were analyzed in one day and in one experiment. Slides were deparaffinized and exposed to heat-induced antigen retrieval for 5 minutes in an autoclave at 121°C in pH 7.8 Tris-EDTA-Citrate buffer prior to incubation with antibody MTC02 (Abcam; 1/450 dilution) detecting a nonglycolizated mitochondrial protein of 60 KD. Bound antibody was visualized using the EnVision Kit (Dako). MTC02 staining was homogenous in the analyzed tissue samples and staining intensity of all cases was semiquantitatively assessed in four categories: negative, weak, moderate and strong immunostaining.

### Statistics

Statistical calculations were performed with JPM 9 software (SAS Institute Inc., NC, USA). Contingency tables and the chi^2^-test were performed to search for associations between molecular parameters and tumor phenotype. Survival curves were calculated according to Kaplan-Meier. The Log-Rank test was applied to detect significant differences between groups. COX proportional hazards regression analysis was performed to test the statistical independence and significance between pathological, molecular and clinical variables.

## Results

### Technical issues

A total of 2,744 of 11,152 (24.6%) tissue spots were non-informative for MTC02 immunohistochemistry due to the complete lack of tissue or absence of unequivocal cancer cells on the respective TMA spots.

### MTC02 immunohistochemistry

**Figure 1:**
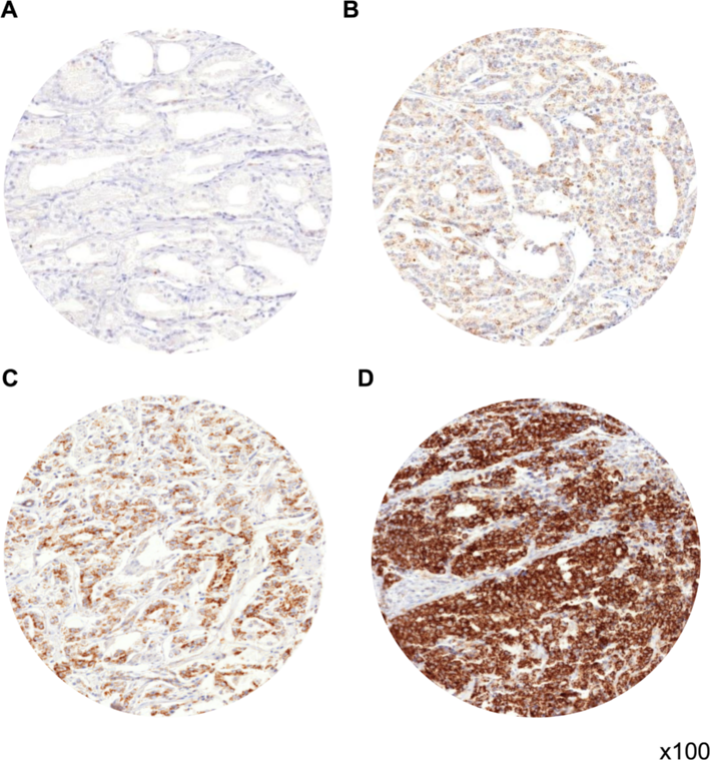
Representative pictures of (A) negative, (B) weak, (C) moderate, and (D) strong MTC02 immunostaining in prostate cancer.

MTC02 immunostaining was located in the cytoplasm of prostate cells. Cancer cells showed higher staining intensities as compared to normal prostate glands. No differences in the staining patter of the different prostate cancer subtypes were observed. In prostate cancer, MTC02 immunostaining was found in 96.5% of the 8,412 analyzable prostate cancers and was considered strong in 46.5%, moderate in 34.6% and weak in 15.4% of tumors. Representative images demonstrating MTC02 expression in prostate cancer tissue are given in Figure 1. Strong MTC02 staining was associated with advanced pathological tumor stage, high Gleason grade, positive nodal involvement (*p* < 0.0001 each), positive surgical margin (*p* = 0.0005), and early PSA recurrence if all cancers were jointly analyzed (*p* < 0.0001).

### Association to cell proliferation

In order to study the impact of mitochondrial content on cell proliferation, we compared MTC02 data with immunohistochemical Ki67 expression that was available from a previous study [33]. We found a strong positive association of MTC02 with Ki67 label index (*p* < 0.0001).

### Association with fusion type prostate cancer

**Figure 2:**
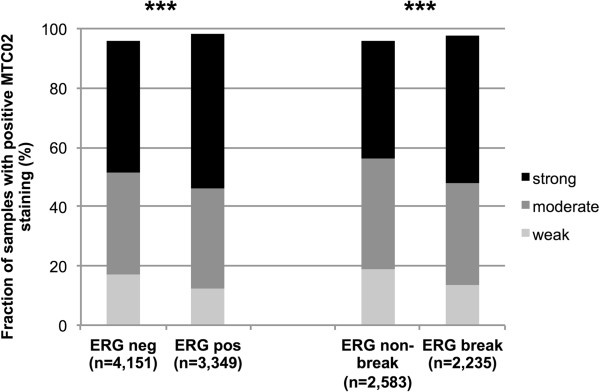
**Relationship of MTC02 expression with *ERG*-fusion status probed by IHC and FISH.** Strong MTC02 immunostaining was slightly more prevalent in *ERG* fusion positive prostate cancers, regardless if the *ERG* status was obtained by IHC or FISH analysis (*p* < 0.0001 each).

To determine whether the mitochondrial content is linked to fusion type prostate cancer, we compared MTC02 staining with the *ERG*-fusion status (obtained by FISH and IHC in 4,818 and 7,500 tumors with MTC02 data) available from our database. Strong MTC02 immunostaining was slightly more prevalent in *ERG* fusion positive prostate cancers, regardless if the *ERG* status was obtained by IHC or FISH analysis (*p* < 0.0001 each; Figure 2). Based on these data, associations with tumor phenotype and clinical cancer features were separately analyzed in the subsets of ERG positive and negative prostate cancers (Tables 2/3). In 4,151 ERG negative cancers, strong MTC02 staining was significantly associated with high preoperative PSA-levels (*p* = 0.0372), advanced pathological tumor stage, high Gleason grade, positive nodal involvement and positive surgical margin status (*p* < 0.0001 each; Table 2). In 3,349 ERG positive prostate cancers, these associations were largely inexistent, although there was still a weak association between MTC02 staining and high Gleason grade (*p* = 0.008; Table 3).

**Table 2:** Associations between MTC02 expression results and ERG negative prostate cancer phenotype

		**MCT02 IHC result**				
**Parameter**	**n evaluable**	**Negative (%)**	**Weak (%)**	**Moderate (%)**	**Strong (%)**	**p value**
**All cancers**	4.15	4.2	17.1	34.2	44.5	
**PSA preoperative**						
<4	423	4.02	18.2	33.57	44.21	0.0372
4-10	2.43	3.88	17.94	35.42	42.76	
10-20	925	5.08	16.11	32.76	46.05	
>20	337	3.56	12.76	31.16	52.52	
**Tumor stage**						
pT2	2.74	4.42	20.02	36.24	39.31	< 0.0001
pT3a	859	4.07	13.62	32.83	49.48	
pT3b	515	3.3	8.16	25.83	62.72	
pT4	26	0	7.69	30.77	61.54	
**Gleason grade**						
≤3 + 3	906	6.62	25.17	38.19	30.0	< 0.0001
3 + 4	2.32	3.67	17.02	36.29	43.02	
4 + 3	685	3.5	11.09	26.86	58.54	
≥4 + 4	226	1.77	3.98	19.91	74.34	
**Lymph node metastasis**						
N0	2.41	4.28	16.09	33.85	45.78	< 0.0001
N+	230	3.04	6.52	23.04	67.39	
**Surgical margin**						
Negative	3.29	4.01	18.09	35.2	42.71	< 0.0001
Positive	783	4.34	13.28	29.89	52.49	

**Table 3:** Associations between MTC02 expression results and ERG positive prostate cancer phenotype

		**MCT02 IHC result**				
**Parameter**	**n evaluable**	**Negative (%)**	**Weak (%)**	**Moderate (%)**	**Strong (%)**	**p value**
**All cancers**	3.35	1.8	12.2	33.8	52.2	
**PSA preoperative**						
<4	451	2.66	11.97	32.59	52.77	0.9613
4-10	2.03	1.73	12.03	34.12	52.12	
10-20	604	1.66	12.75	33.44	52.15	
>20	218	1.83	13.3	35.78	49.08	
**Tumor stage**						
pT2	1.980	1.92	11.31	34.55	52.22	0.5463
pT3a	902	1.77	13.86	33.81	50.55	
pT3b	427	1.64	12.41	30.68	55.27	
pT4	22	0	18.18	36.36	45.45	
**Gleason grade**						
≤3 + 3	745	3.36	10.07	36.64	49.93	0.008
3 + 4	1.98	1.26	12.32	33.01	53.41	
4 + 3	485	2.06	14.85	33.81	49.28	
≥4 + 4	114	0.88	13.16	32.46	53.51	
**Lymph node metastasis**						
N0	1.9	1.63	12.2	34.56	51.6	0.7185
N+	192	1.04	14.58	32.81	51.56	
**Surgical margin**						
Negative	2.62	1.83	12.48	33.73	51.96	0.825
Positive	670	1.94	11.19	34.63	52.24	

### Relationship with key genomic deletions associated with distinct subgroups of prostate cancers

**Figure 3:**
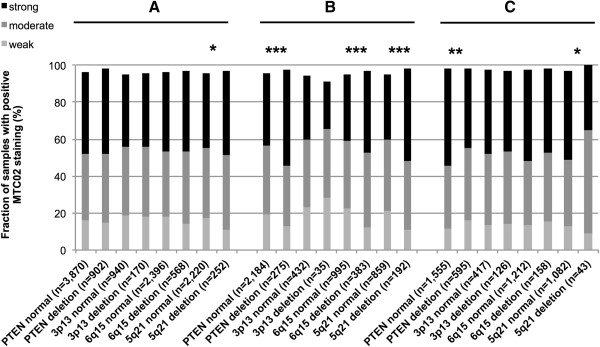
**Relationship between MTC02 expression and deletions of *PTEN*, 3p13, 6q15 and 5q21 probed by FISH analysis. (A)** Association between MTC02 expression and deletions of *PTEN* (*p* = 0.0596), 3p13 (*p* = 0.0989), 6q15 (*p* = 0.0867) and 5q21 (*p = 0.0253) in all prostate cancers. Relationship of MTC02 expression with **(B)** deletions of *PTEN* (***p < 0.0001), 3p13 (*p* = 0.641), 6q15 (***p < 0.0001) and 5q21 (***p < 0.0001) in the subset of ERG negative prostate cancers and with **(C)** deletions of *PTEN* (**p = 0.0004), 3p13 (*p* = 0.9491), 6q15 (*p* = 0.7544) and 5q21 (*p = 0.0408) in the subset of ERG positive cancers.

Earlier studies had provided evidence for distinct molecular subgroups of prostate cancers defined by fusion status and several genomic deletions. Others and us had described strong associations between deletions of *PTEN* and 3p13 and ERG positive cancers and between deletions of 5q21 and 6q15 and ERG negative tumors [30-32,34-36]. To study, whether or not some of these subgroups may have a particularly high mitochondrial content, MTC02 immunostaining was compared with preexisting deletion results. Interestingly, mitochondrial content was largely unrelated to all analyzed chromosomal deletions if all tumors were analyzed (Figure 3A) while there were reciprocal statistically significant findings in the subgroups of ERG positive and ERG negative cancers. In ERG negative cancers, most deletions (*PTEN*, 5q, 6q; *p* < 0.0001 each; Figure 3B) were significantly associated with high mitochondrial content, while there was a tendency towards lower mitochondrial content in ERG positive cancers harboring deletions (Figure 3C). This tendency did, however, reach significance only for deletions of *PTEN* (*p* = 0.0004) and 5q (*p* = 0.0408).

### Prognostic impact

**Figure 4:**
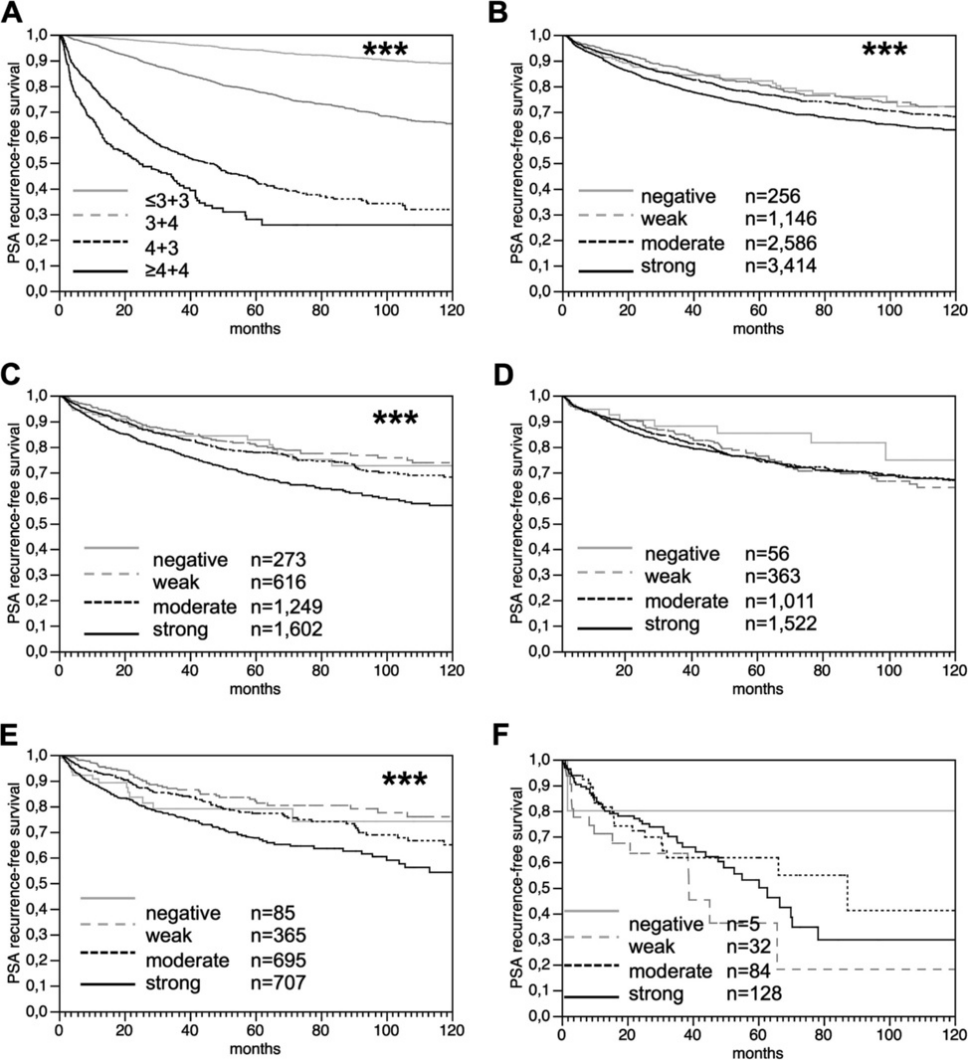
**The prognostic impact of MTC02 expression in prostate cancer.** The prognostic role of Gleason grade is given for this patient subset in order to demonstrate the overall validity of our follow-up data (***p < 0.0001) **(A)**. Association of MTC02 immunostaining with biochemical recurrence in **(B)** all prostate cancers (***p < 0.0001; n = 7,402), **(C)** in the subset of ERG negative cancers (***p < 0.0001; n = 3,616), **(D)** in the subset of ERG positive cancers (*p* = 0.7598; n = 2,952), **(E)** ERG negative prostate cancers lacking *PTEN* deletions (***p < 0.0001; n = 1,852), and **(F)** ERG negative prostate cancers harboring *PTEN* deletion (*p* = 0.2367; n = 249).

Follow-up data were available from 7,402 patients with data on mitochondrial content. The prognostic role of Gleason grade was plotted for this patient cohort in order to demonstrate the overall validity of our follow-up data (*p* < 0.0001; Figure 4A). High MTC02 immunostaining was related to early biochemical recurrence if all cancers were analyzed (*p* < 0.0001; Figure 4B). A subset analysis revealed, that this association was purely driven by ERG negative cancers (*p* < 0.0001; Figure 4C) while the mitochondrial content was unrelated to PSA recurrence in ERG positive cancers (*p* = 0.7598, Figure 4D). A refined analysis further revealed that the prognostic relevance of MTC02 was limited the 1,852 ERG-negative cancers lacking *PTEN* deletion (*p* < 0.0001; Figure 4E), while there was no effect in 249 ERG negative cancers harboring *PTEN* deletions (*p* = 0.2367; Figure 4F).

### Multivariate analysis

Four multivariate analyses were performed evaluating the clinical relevance of MTC02 immunostaining in different scenarios (Table 4A-C). Analysis #1 employed all post-operatively available parameters including pT, pN, margin status, pre-operative PSA value and Gleason grade obtained on the resected prostate. Scenario #2 included all postoperatively available parameters with the exception of nodal status. The rational for this approach was that lymphadenectomy is not a routine procedure in the surgical therapy of prostate cancer and that excluding pN in multivariate analysis increases case numbers. The remaining two scenarios tried to better model the pre-operative situation. Scenario #3 included the mitochondrial content, pre-operative PSA, clinical stage (cT) and the Gleason grade obtained on the prostatectomy specimen. Because the post-operative Gleason grade varies from the pre-operative Gleason grade, another multivariate analysis (#4) was added. In scenario #4, the pre-operative Gleason grade obtained on the original biopsy was combined with pre-operative PSA, clinical stage and MTC02 staining. The diverse multivariate analyses suggest a possible independent prognostic impact of MTC02 immunostaining in a preoperative setting, especially in ERG negative cancers (Table 4).

**Table 4:** Multivariate analysis including MTC02 expression status in (A) all cancers, (B) ERG negative and (C) ERG positive prostate cancers

A								
**Scenario (n)**	**preoperative PSA-level**	**pT stage**	**cT stage**	**Gleason grade prostatectomy**	**Biopsy gleason grade**	**N status**	**R status**	**MTC02 expression**
1 (4,433)	< 0.0001	< 0.0001		< 0.0001		< 0.0001	< 0.0001	0.656
2 (7,226)	< 0.0001	< 0.0001		< 0.0001			< 0.0001	0.8726
3 (7,078)	< 0.0001		< 0.0001	< 0.0001				0.1282
4 (6,974)	< 0.0001		< 0.0001		< 0.0001			0.0007
B								
1 (2,220)	0.0026	< 0.0001		< 0.0001		< 0.0001	0.0063	0.7664
2 (3,528)	< 0.0001	< 0.0001		< 0.0001			0.0002	0.8366
3 (3,489)	< 0.0001		< 0.0001	< 0.0001				0.7614
4 (3,442)	< 0.0001		< 0.0001		< 0.0001			0.0384
C								
1 (1,791)	0.0117	< 0.0001		< 0.0001		0.0118	0.003	0.7873
2 (2,884)	0.0002	< 0.0001		< 0.0001			< 0.0001	0.8902
3 (2,798)	< 0.0001		< 0.0001	< 0.0001				0.2321
4 (2,752)	< 0.0001		< 0.0001		< 0.0001			0.3955

## Discussion

The results of our study show, that the mitochondria content is tightly linked to various pathological, molecular features of prostate cancer. This data highlight the prominent importance of mitochondrial function for prostate cancer development and progression.

Immunohistochemical detection of a 60 KDa non-glycosylated protein component of mitochondria was utilized in this project to quantitate mitochondria in cancer cells on TMAs. The TMA approach is optimal for the identification of subtle staining differences of proteins that are abundantly present in cancer, such as mitochondrial components, because TMAs enable maximal experimental standardization. In this study, more than 10,000 prostate cancer specimens were analyzed in one day in one experiment using one set of reagents at identical concentrations, temperatures and exposure times. Moreover, all TMA sections were cut on one day immediately before staining in order to avoid unequal decay of a tissues reactivity to antibody binding [37]. Finally, one pathologist interpreted all immunostainings at one day to enable maximal standardization of staining interpretation. In earlier studies, our TMA enabled us to validate several biomarkers with importance for prostate cancer, such as p53 expression [38], *PTEN* inactivation [32], CRISP3 overexpression [39] or deletions at 6q15 [31] and 5q21 [30].

The data derived from this approach demonstrate a marked increase of mitochondria content from normal prostate epithelial cells to cancer cells. A further increase was observed with increasing tumor grade and stage, suggesting that higher numbers of mitochondria are necessary or supportive for cancer development and progression. This is also supported by the observation that the mitochondrial content was linked to increased cell proliferation. Our findings are consistent with recent studies suggesting a prominent role of mitochondria content in cancer. For example, Ambrosini-Apaltro et al. [12] detected oncocytic, mitochondrion-rich modifications in adenocarcinoma cells after radiochemotherapy and Ragazzi et al. [26] described a link between mitochondrion-rich and undifferentiated breast cancers. Despite the early belief that cancer metabolism is primitive and inefficient, it has now become evident that cancer cells actively reprogram their metabolism activity [40]. Adaptation of cellular metabolism towards macromolecular synthesis is critical to supplying sufficient amounts of nucleotides, proteins, and lipids for cell growth and proliferation, which are fundamental to cell growth and proliferation [40]. Accordingly, previous studies described interactions between the mitochondrial metabolism and the activity of growth signaling pathways involving key human oncogenes such as Myc, Ras, Akt and phosphoinositide 3-kinase (Pi3K) [41-43]. Activated PI3K/Akt leads to enhanced glucose uptake and glycolysis [44,45] by induction of glucose transporters, mitochondrial enzymes involved in the glycolytic metabolism and glucose carbon flux into biosynthetic pathways [46-49]. Downstream of PI3K/Akt, the well-characterized cell growth regulator mTORC1 also has many effects intertwined with mitochondrial metabolism [50-52]. Taken together, these findings demonstrate that the reprogramming of mitochondrial metabolism is a central aspect of PI3K/Akt associated oncogenic activity.

The large number of tumors analyzed in this study enabled us to separately analyze cancer subgroups defined by molecular features, the most common of which is the *TMPRSS2:ERG* gene fusion. Gene fusions involving the androgen-regulated gene *TMPRSS2* and *ERG*, a member of the *ETS* family of transcription factors, occur in about 50% of prostate cancers, especially in young patients, and result in strong ERG protein overexpression [53-55]. Our data demonstrate that high mitochondrial content is significantly linked to fusion type prostate cancer. Finding this association by two independent approaches for *ERG* fusion detection (IHC/FISH) largely excludes a false positive association due to inefficient immunostaining for both MTC02 and ERG in a subset of damaged non-reactive tissues. This finding strongly argues for generally increased energy demands of ERG positive as compared to ERG negative cells. ERG expression causes massive deregulation of the global expression patterns in prostate cells. Several studies analyzing the transcriptomes of *ERG* positive and *ERG* negative tumors revealed that multiple energy-consumptive signaling pathways are activated as a result of ERG expression, including ER-, TGF-ß, WNT, PI3K/Akt and Myc signaling [56-60] all of which involve multiple ATPases and ATP-dependent kinases. Particularly PI3K/Akt and Myc signaling also directly activates glycolysis [43] and induces transcription of numerous glycolytic enzymes [4] in cancer cells.

That mitochondrial content has a different role and function in ERG positive and ERG negative cancers is further supported by our ERG-stratified analysis of disease outcome. Mitochondrial content had a prognostic role in ERG negative but not in ERG positive cancers. This striking difference may be caused by the substantial increase of cellular mitochondrial content by ERG rearrangements, which by themselves do not have any prognostic impact on prostate cancers. The magnitude of ERG-induced molecular and cellular changes, at least most of which are unrelated to cancer progression, may lead to an increased mitochondrial content in “fusion-type” prostate cancers, that masks demand for higher mitochondria content caused by specific molecular “progression events” requiring more mitochondrial function. The strong prognostic impact of mitochondria content in ERG negative prostate cancers fits well with models suggesting, that in a surrounding with low mitochondria content, “progression events” requiring more mitochondrial function would rather lead to a detectable increase of the mitochondria count, than in an environment with high mitochondria content.

Deletions of *PTEN*, 5q21 and 6q15 represent such “progression events” in prostate cancer as all of them are strongly linked to tumor growths and adverse clinical features. It seems likely that a shortage of nutrients and oxygen typically occurring during tumor expansion will eventually trigger additional adaptation steps, and increase of the mitochondrial content might be one of these. That such an increase of the mitochondrial content was not observed for 3p13 deletions may be due to the low number of analyzed ERG negative tumors for this deletion. Alternatively, it might be due to the small number of genes affected by these small 3p13 deletions, none of which may lead to additional “energy demand” in case of inactivation. A role of *PTEN* inactivation as a “progression event” associated with higher requirements for mitochondrial function is further supported by the observation that high mitochondrial content loses its prognostic relevance in *PTEN* deleted ERG negative cancers.

It is of note that the relationship between all analyzed deletions (*PTEN*, 3p13, 5q21, 6q15) and the mitochondria content tended to invert within ERG-positive cancers. The causes for this observation cannot be deducted from our data. It might be speculated, that non-vital ERG induced mitochondria production is restrained under a different cellular environment driving towards tumor progression including more rapid tumor cell growth. More specifically, it may be possible that specific molecular events caused by chromosomal deletions interfere with ERG induced general upregulation of number of mitochondria. It has indeed been shown, that *PTEN* inactivation can directly trigger both glycolysis [61] and mitochondrial respiratory capacity [62] through AKT/mTOR signaling activation.

The marked prognostic relevance of mitochondrial abundance found in the subset of ERG negative cancers may suggest “mitochondria content” as a biomarker with potential clinical utility. This notion is further supported by the fact that the prognostic impact of mitochondria content was found on a TMA containing just one 0.6 mm cancer sample per patient. This approach of analyzing molecular features closely models the molecular analyses of core needle biopsies where comparable amounts of tissues are evaluated. Various models of multivariate analyses applied in this project indeed suggested an independent predictive role of mitochondria content for prognosis if only parameters were utilized that are available before radical prostatectomy. These data must be interpreted with caution, however, because the MTC02 immunostaining was done on tissue from radical prostatectomies and not on the core needle biopsies that were used to determine the preoperative Gleason grading. It is obvious, that potential prognostic biomarkers should be evaluated on preoperative needle biopsies but from a practical point of view such analyses are hardly feasible. This is because needle biopsies are usually done at many different facilities and not accessible for studies. Moreover, if such precious core needle biopsies were available, they would be exhausted after only few studies. Independent of this, it might be rewarding to further consider mitochondria content as a potential feature in multiparametric prognostic prostate cancer tests.

In summary, the results of our study highlight a different role of mitochondrial content in *ERG* fusion-positive and -negative cancers and identify “mitochondrial abundance” as a potential prognostic feature in ERG-negative cancers. Strong associations between chromosomal deletions and the cellular mitochondrial content further highlight the important role of mitochondria content as an adaptation process during cancer progression.

## Competing interests

The authors declare that they have no competing interest.

## Authors’ contribution

GS, TS, JI, SS, RS and SM designed research; KG, KJ, MCT, CK, WW, AQ and MA performed the experiments and analyzed the data. GS, RS, KG, HH and MG wrote the manuscript. All authors read and approved the manuscript.
